# Phase separation and biomolecular condensate formation drive plant endomembrane and autophagy crosstalk

**DOI:** 10.1093/jxb/eraf269

**Published:** 2025-06-25

**Authors:** Chiamaka Linda Mgbechidinma, Junfeng Cao, Liwen Jiang

**Affiliations:** School of Life Sciences, State Key Laboratory of Agrobiotechnology, The Chinese University of Hong Kong, Shatin, Hong Kong, China; AoE Centre for Organelle Biogenesis and Function, Centre for Cell and Developmental Biology, The Chinese University of Hong Kong, Shatin, Hong Kong, China; School of Life Sciences, State Key Laboratory of Agrobiotechnology, The Chinese University of Hong Kong, Shatin, Hong Kong, China; AoE Centre for Organelle Biogenesis and Function, Centre for Cell and Developmental Biology, The Chinese University of Hong Kong, Shatin, Hong Kong, China; School of Life Sciences, State Key Laboratory of Agrobiotechnology, The Chinese University of Hong Kong, Shatin, Hong Kong, China; AoE Centre for Organelle Biogenesis and Function, Centre for Cell and Developmental Biology, The Chinese University of Hong Kong, Shatin, Hong Kong, China; Institute of Plant Molecular Biology and Agricultural Biotechnology, The Chinese University of Hong Kong, Shatin, Hong Kong, China; CUHK Shenzhen Research Institute, Shenzhen 518057, China; Michigan State University, USA

**Keywords:** Autophagosome, autophagy, biomolecular condensates, endomembrane system, liquid–liquid phase separation, membrane trafficking, multivesicular body, vacuole

## Abstract

Like other eukaryotes, plants are a rich hub of proteins, lipids, and nucleic acid biomolecules that undergo liquid–liquid phase separation to form liquid-like biomolecular condensates that facilitate diverse cellular functions, especially upon biotic and abiotic stresses. Current plant-related research highlights the emerging role of biomolecular condensates in stress sensing, modulation, and response as an intricate mechanism for rapid and efficient stress adaptation. The cellular functions of condensates and their localization emphasize the importance of endomembrane systems in bridging the understanding of membrane-bound and membrane-less organelles and their compartmentalization. This review provides an overview of the recent updates and findings in plant phase separation and biomolecular condensate formation. With the increasing evidence of research pointing to a link between membrane-less condensates, autophagy, and the endomembrane system, we discuss the crosstalk between the multivesicular body (MVB), autophagosome, and vacuole. We also elaborate on the functional and regulatory roles of biomolecular condensates in plant autophagosome formation at the early and late stages. Finally, we provide insights for future investigations on plant cellular biomolecular condensates to pave the way for new frontiers of studies in improving agricultural plant yield, resilience, and other biotechnological applications.

## Teaser

Endomembrane and autophagy pathways are intricately linked in plant cellular homeostasis, with biomolecular condensates formed through liquid–liquid phase separation driving the crosstalk.

## Introduction

**Fig. 1.**
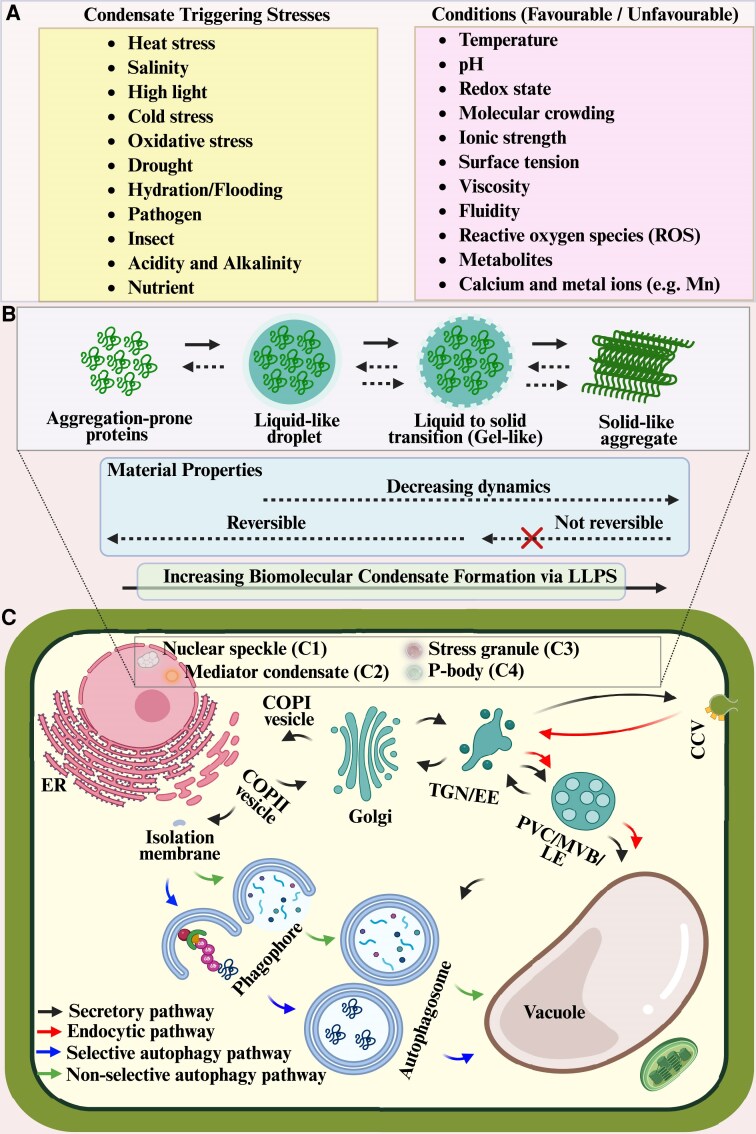
Compartmentalization of membrane-bound and membrane-less organelles in the plant endomembrane system and autophagic pathway. (A) Diverse environmental signals and conditions trigger intercellular modifications that may initiate liquid–liquid phase separation (LLPS) and its transition within and across several cellular compartments. (B) LLPS is a stage in the phase transition process during biomolecular condensate formation. Cells under different conditions undergo phase transition processes. (C) Common cellular condensates C1–C4 formed from aggregation-prone proteins, and subsequent LLPS are localized in the nucleus (nuclear speckle, C1; and Mediator condensate, C2) and cytoplasm (stress granule, C3; and P-body C4). The endomembrane system and autophagy are intricately linked through the coordinated activity of several organelles including the endoplasmic reticulum (ER), Golgi apparatus, *trans*-Golgi network (TGN) or early endosome (EE), multivesicular body (MVB) or pre-vacuolar compartment (PVC) or late endosome (LE), and the vacuole, which characterize the secretory pathway. Clathrin-coated vesicles (CCVs) play a crucial role in the endocytic pathway by internalizing plasma membrane components and extracellular materials into CCVs, facilitating their cellular uptake as cargo to the sorting endosome for recycling back to the plasma membrane via recycling endosomes or targeted degradation channelled to the vacuole. Coat protein complex II (COP II) vesicles transport cargo (primarily proteins) from the ER to the Golgi. In contrast, COP I vesicles mediate the retrograde transport from the Golgi to the ER, ensuring proper organelle function. Some specific COPII components (Sar1 and Sec23 proteins) are involved in the conventional ER–Golgi trafficking and also regulate plant autophagic flux by interacting with the core autophagy machinery. Remarkably, these vesicular trafficking machineries have been reported to provide membrane sources for autophagosome formation. The Golgi, TGN, and MVB contribute to the sorting and delivery of autophagic cargo, with the vacuole serving as the final destination for degradation, highlighting the dynamic interplay between these compartments in maintaining cellular homeostasis. Within plant cells, the MVB, autophagosome, and vacuole are closely related endomembrane organelles. Notably, the autophagy pathways can be bulk or selective depending on the sequestered cargoes. Created in BioRender. Mgbechidinma *et al*. (2025) https://BioRender.com/ai1pc3r.

Liquid–liquid phase separation (LLPS) and the resultant formation of biomolecular condensates as an integral part of plant membrane trafficking are gaining increasing attention in recent research, owing to the need to understand how compartmentalized membrane-bound organelles interact or co-exist with membrane-less organelles. While several reversible and non-reversible phase transition processes are inherent in cells, in response to several environmental cues (Fig. 1A, B), these organelles coordinate several spatial and temporal pathways within (Fig. 1C), having proteins as the major building block. Proteins with repetitive interaction motifs or intrinsically disordered regions (IDRs) consisting of low-complexity domains and highly biased amino acid compositions through multivalent interactions, such as van der Waals interactions, electrostatic interactions, hydrophobic interactions, and cation–π interactions, trigger LLPS (Poudyal *et al.*, 2023; Koja *et al.*, 2024). Dai *et al*. (2023) have detailed the principles of these interactions. Unique to these types of proteins are those containing the prion-like domains (PrLDs), capable of regulating plant stress responses via their scaffolds that recruit other proteins through a heterotypic phase separation to form condensates (Hutin *et al.*, 2023; Legen *et al.*, 2024).

One of the fundamental concepts in understanding how intrinsically disordered proteins (IDPs) undergo phase separation through homotypic interactions to form biomolecular condensates is the ‘stickers-and-spacers’ model. The model, as commonly proposed in phase separation articles, is a function of specialized amino acids (Dai *et al.*, 2023; Fernando *et al.*, 2024), whereby the stickers aiding cohesive interactions consist of aromatic residues (tyrosine and phenylalanine), along with charged residues within the RNA-binding domains (arginine and lysine). Meanwhile, spacers that provide a structural sticker scaffold consist of glycine (for liquidity), glutamine, and serine (for hardening). Stickers drive multivalent interactions, while spacers enhance the liquid-like and fluid properties of phase-separated condensates by providing flexibility and spacing between the stickers (Jeon *et al.*, 2025). While sequences rich in glycine and glutamine possess physical properties that promote phase separation, the presence of more glutamic acid, serine, and proline indicates more gel-like or solid-like condensates, with reduced reversibility and dynamics (Fig. 1B). Thus, resolving the composition and organization of the stickers and spacers could be a rapid mechanism for elucidating the physicochemical dynamics and phase transition of biomolecular condensates. Prioritizing the translational applications of the sticker-and-spacer model has revealed its potential for engineering cellular control systems in synthetic biomolecular condensate design following the modular principles (Dai *et al.*, 2023). However, delving into the biophysical complexities of the role of stickers in driving condensate formation, recent research has shown the importance of discretized interaction energies and spacer dynamics, offering a more nuanced understanding of phase separation mechanisms. As such, advancing the model by integrating a multiscale computational framework to simulate the dynamic assembly and disassembly of IDPs, such as FUS (Fused in Sarcoma), emphasizes the stochastic nature of sticker–sticker interactions and their modulation by environmental factors (Fernando *et al.*, 2024). This reinforces the importance of integrating computational, experimental, and theoretical approaches to elucidate biomolecular condensate formation fully. In mammals, the stickers-and-spacers model is widely applied to understand membraneless organelles such as nucleoli and P-bodies. Plant studies have shown the relevance of the model in hydration-dependent phase separation during seed germination, as the glutamine, proline, and serine (QPS)-rich PrLD spaced aromatic tyrosine residues along its sequences (stickers) (Dorone *et al.*, 2021). Increasing QPS PrLD stickiness induces the gelation of an uncharacterized prion-like protein FLOE1, suggesting that sticker number alteration can result in a liquid-to-gel transition, while the increasing multivalency drives gelation to solid-like assemblies. Thus, the balance between sticker-mediated attractions and spacer-mediated solvation determines whether phase separation occurs. This process can be validated through experimental techniques such as single-molecule fluorescence, FRAP (fluorescence recovery after photobleaching), and EM (Dorone *et al.*, 2021).

Alternative models, such as the patchy particle model and multivalency-mediated phase separation, also provide a complementary approach for understanding condensate formation through the role of discrete interaction sites (patches) and network connectivity by stereospecific interactions, such as those between folded protein domains or RNA-binding motifs, rather than relying solely on stickers and spacers (Dar *et al*., 2024; J. Kim *et al*., 2024). While the patchy particle model has not been explicitly documented in plant-specific studies, it is a powerful computational framework that can be used in plant biomolecular condensate research to simulate the formation, dynamics, and regulation of condensates, offering insights into their assembly mechanisms, response to environmental cues, and how the biomolecules modulate their properties. Multivalency-mediated phase separation involves the separation of the homogeneous mixture of biomolecules into distinct phases (dense and dilute) driven by multiple interaction sites on the molecules, thus suggesting the interacting ability of a molecule. For instance, a protein can interact with multiple partners (RNA or other proteins) at once through several binding sites or domains, creating a network of interactions that defines the term ‘multivalency’ (Hutin *et al.*, 2023; Jeon *et al.*, 2025). In plant studies, multivalency is detected through the presence of non-covalent and hydrophobic interactions that can be disrupted by the chemical 1,6-hexanediol, the presence of specific RNA structures (G-quadruples that promote gel-like phase separation), the presence of head-to-tail polymerization (where specific domain interactions lead to large multivalent complex assembly), the presence of methylated arginine and acetylated lysine through post-translational modification (PTM), and the occurrence of domain–domain interactions (RNA-binding proteins with domains such as RGG, RRM, and PrLD), which promotes multivalent interactions and phase separation (Wang *et al.*, 2022; Legen *et al.*, 2024; Poveda-Cuevas *et al.*, 2024; Tang *et al.*, 2024; Jeon *et al.*, 2025; Lei *et al.*, 2025).

The concept of LLPS transcends beyond plants, as it has been actively investigated in prokaryotes (Hoang *et al.*, 2024) and other eukaryotes (yeast and mammals) (Franzmann *et al.*, 2018; Wang *et al.*, 2021), mostly in proximity to cellular membranes aiding bending, tethering, sequestration, scission, and exclusion—known membrane trafficking mechanisms (Prinz *et al.*, 2020; Wang *et al.*, 2024). Cell type-specific membrane-associated condensates contribute to specialized cellular properties and functions owing to their cohesive forces at the interface between a condensate and its surrounding aqueous environment (surface tension) as well as the interaction between a condensate and a membrane surface, where the condensate spreads over or adheres to the membrane (wetting), even at membrane contact sites (Prinz *et al.*, 2020; Poveda-Cuevas *et al.*, 2024; Wang *et al.*, 2024). Based on the molecular characteristics, condensate–membrane interactions can be categorized into three types: (i) dewetted, where the condensate remains spherical with limited membrane contact; (ii) partial wetting, characterized by an intermediate contact angle (between 0° and 180°) with partial spreading; and (iii) complete wetting, where the condensate fully spreads across the membrane surface as a thin layer (Lalchand *et al.*, 2025). Notably, wetting occurs when the surface tension of the condensate and the surface properties of the membrane favour contact, reducing the overall free energy, a common phenomenon in condensates localized in the plasma membrane (PM) and endomembrane organelles (Kusumaatmaja *et al.*, 2021; Dragwidge *et al.*, 2024; Tang *et al.*, 2024; Wang *et al.*, 2024). Although not verified in plants, the phase-separating IDR and the cytosolic proline-rich domain (PRD) of the single-pass transmembrane protein SCOTIN, detected in the endoplasmic reticulum (ER) and endosome membranes, regulates their tethering and perinuclear endosome positioning for proper endosome trafficking via a homotypic interaction (Yun *et al.*, 2023). Other IDRs that have not been confirmed for their condensate-forming ability are shown to mediate membrane tethering. IDRs in the Oxysterol-binding protein (OSBP) and OSBP-related proteins (ORPs), especially ORP5 and ORP8, within the ER–PM membrane contact sites, interact electrostatically to tether these membranes (Jamecna *et al.*, 2019). The IDRs possessing membrane tethering plasticity and flexibility account for how the VAP-A universal ER receptor interacts with numerous targets in the membrane contact sites between the stable ER–mitochondria and short-lived ER–*trans*-Golgi network (TGN) membranes, enabling lipid transfer by interacting with phosphatase-interacting protein 51 (PTPIP51), channel-like protein (VPS31A), OSBP, and the ceramide-transfer protein (CERT) (Subra *et al.*, 2023). Besides controlling the protein orientations and aiding organelle tethering, the interaction between condensates and membranes at contact sites (areas of organelle close proximity) may facilitate cellular communication or material exchange, a research gap in the crosstalk between plant condensates and membrane contact sites.

Among the several organelles involved in plant membrane trafficking pathways, the endomembrane organelle the ER stands out as the site for condensate biogenesis, dynamics, and assembly (Wang *et al.*, 2021; Tang *et al.*, 2024). This accounts for the first line of crosstalk between cellular biomolecular condensate and autophagy, an evolutionarily conserved mechanistic recycling and reuse process (Zhuang *et al.*, 2024). Well studied in plants is macroautophagy (hereafter referred to as autophagy), known for its characteristic double-membered autophagosome, which originates from an ER-derived isolation membrane before being trafficked and delivered to the vacuole, a potential autophagosome formation site through LLPS (Jiang *et al.*, 2024; Mosesso *et al.*, 2024; Yan *et al.*, 2024; Zhang *et al.*, 2025). Upon liquidity through continuous phosphorylation and dephosphorylation, the ATG1 complex-derived PAS (pre-autophagosomal structure) can be tethered to the vacuolar membrane Vac8 through ATG13 interaction in yeast (Fujioka *et al.*, 2020). In the biophysics of membrane remodelling, the C-terminal IDR of the ER-phagy receptor (FAM134B) autonomously senses and generates membrane curvature, while its tethering to the reticulon homology domain (RHD) enhances curvature-driven protein clustering and accelerates membrane budding (Poveda-Cuevas *et al.*, 2024). The interplay between IDR dynamics and RHD scaffolding mechanistically underpins selective ER-phagy by modulating membrane topology and autophagosome formation. Hence, condensate-prone proteins can regulate membrane shapes and organelle remodelling through signal transduction, scaffolding, and vesicular trafficking. Other condensate-interacting cellular organelles in plants include the PM, nucleus, endosome, vacuole, and chloroplast. The LLPS thermodynamics and overall phase transition are dependent on the type and concentrations of macromolecules (membrane-associated proteins, soluble proteins, RNA, lipid-binding domains, lipids, and integral cellular lipids), as well as physical factors such as temperature, osmolarity, and pH, as featured in Fig. 1A (Franzmann *et al.*, 2018; Dai *et al.*, 2023; Xie *et al.*, 2024; Jeon *et al.*, 2025; Licheva *et al.*, 2025; Nair *et al.*, 2025). It remains unknown how a shift in the composition and concentration of these biomolecules could influence the induction or inhibition of condensate formation. While there is increasing interest in proteins in relation to biomolecular condensate and their signalling transduction alongside interaction with cellular organelles, investigations into the RNA-binding domains can be instrumental in understanding how structured RNA, serving as scaffolds, enables nucleation during biomolecular condensate formation. The RNA recognition motif (RRM), K homology domain, zinc finger domain, DEAD/DEAH box helicase domain, and hinge region (LHP1 specific), commonly found in nuclear condensates, dicing bodies, processing bodies (P-bodies), and stress granules, are examples of RNA-binding domains that can be investigated. Concentration is a critical determinant of condensate formation, with fewer RNAs being favourable, whereas higher concentrations of RNAs are inhibitory, a process known to support the concept of transcription bursts (Dai *et al.*, 2023; Legen *et al.*, 2024; Ryu *et al.*, 2024). RNA and protein interactions during LLPS are charge dependent, and a typical RNA-enriched condensate is the stress granules (Franzmann *et al.*, 2018). Notably, experimental investigations into the composition, localization, and translocation of plant condensates within and across several cellular compartments, forming a crosstalk between varying signal transduction networks and pathways, remains an area of active research (Kusumaatmaja *et al.*, 2021; Dragwidge *et al.*, 2024; Legen *et al.*, 2024; P. Li *et al*., 2024; Tang *et al.*, 2024; Wang *et al.*, 2024; Yan *et al.*, 2024; Larran *et al.*, 2025; Lei *et al.*, 2025).

Under stress conditions, condensate assembly or disruption alters plant tolerance and development by destabilizing cellular homeostasis and impairing protein trafficking and degradation. This highlights the critical role of condensates in plant survival. Among known cellular processes, endomembrane trafficking relies on condensates to regulate vesicle formation and transport, while autophagy machineries employ condensates to regulate autophagosome formation, cargo delivery, and degradation under stress. These biological functions are critical for plant cellular homeostasis. Condensates, such as those formed by the TPLATE complex (AtEH1 and AtEH2), are crucial for clathrin-mediated endocytosis. For instance, AtEH1 mutation abolishes condensate formation in a manner that disrupts subcellular localization and function of the protein (Dragwidge *et al.*, 2024). AtEH1 condensates nucleate on the PM via interactions with anionic phospholipids [e.g. phosphatidic acid and phosphatidylinositol-4-phosphate (PI4P)] to recruit clathrin and endocytic accessory proteins. Mutation of the EH domains or IDR1 of AtEH1 abolishes condensate formation, leading to defective clathrin lattice assembly, delayed vesicle scission, and reduced endocytic internalization (Dragwidge *et al.*, 2024). This disrupts the dynamic recruitment of proteins required for membrane bending and vesicle formation, stalling endocytosis at its earliest stages. The IDR1 of AtEH1 regulates the physical properties of condensates (e.g. viscosity, fusion dynamics, and recovery kinetics). Mutations that weaken the aromatic residue interactions (e.g. phenylalanine and tyrosine to serine: YF>S) or strengthen them (e.g. phenylalanine and tyrosine to tryptophan: YF>W) perturb phase separation, resulting in abnormal sphericity, altered fluorescence recovery (FRAP), and impaired molecular exchange within the condensates (Dragwidge *et al.*, 2024). These changes destabilize the balance between solid-like and liquid-like network assemblies that are critical for endocytic efficiency. Disrupted AtEH1 condensates lead to diffuse cytosolic distribution of clathrin, TPLATE, and dynamin proteins (endocytic machinery), which prevents their ordered assembly into functional complexes. The physiological consequences of the loss of condensate-driven endocytosis manifest in developmental defects, which result in delayed root gravitropism and male sterility, as observed in the mutant plants that exhibited impaired pollen viability (Dragwidge *et al.*, 2024). The disrupted membrane trafficking and organelle remodelling highlight the systemic role of AtEH1 condensates in plant growth and reproduction. This unveils the roles of biomolecular condensate in modulating plant endocytosis dynamics, trafficking efficiency, and plant responsiveness to abiotic and biotic stresses. Salt stress induces ion imbalance and osmotic stress, triggering cellular endomembrane trafficking and autophagy. The loss of the transcriptional regulator SEUSS (SEU) condensation in Arabidopsis compromises the expression of osmotic stress tolerance genes (Wang *et al.*, 2022). FREE1 condensates are essential for plant endosomal membrane scission during multivesicular body (MVB) biogenesis (Wang *et al*., 2024); however, phosphorylation of FREE1 by SALT OVERLY SENSITIVE2 (SOS2) impairs MVB trafficking and MVB–vacuole fusion upon salt stress, resulting in increased accumulation of compartmentalized sodium ions (Na^+^) within the fragmented vacuoles to reduce toxicity due to ion imbalance (Liu *et al.*, 2025). Thus, FREE1 condensates may play a crucial role in vesicle formation needed in autophagy, especially in the context of amphisomes (MVB–autophagosome fusion). Under abiotic stresses such as heat, plants undergo LLPS to form stress granules, where autophagy machineries for initiation, expansion, and maturation sequester to facilitate stress recovery, as seen in the delayed activation of autophagy in stress granule-deficient (*ubp1abc*) mutants (X. Li *et al*., 2024). Calcium-sensing proteins such as CALMODULIN-LIKE 38 (CML38), requiring the AAA+-ATPase CELL DIVISION CYCLE 48A, are reported to degrade stress granules, a type of SUPPRESSOR OF GENE SILENCING 3 condensate, during hypoxia (Field *et al.*, 2021). Therefore, when CML38 is impaired, plants may struggle to break down these granules, leading to abnormal RNA regulatory processes. Notwithstanding, a recent study has shown that the genetically encoded X-E3TCD1 fusion protein (Teosinte branched 1) can be used for targeted protein degradation of endogenous condensation-prone proteins (such as native TB1 protein), leading to improved rice tiller number (Luo *et al.*, 2025). This represents a promising condensate-related transgene-based approach for optimal crop performance devoid of small molecules, antibodies, or genetic knockin fusion tags. It remains an issue of debate whether the presence or disruption of condensates is favourable for plant growth and development or whether the functional benefit of condensates is stress type dependent. Most studies on biomolecular condensate have largely reported on its role in enhancing plant survival. It is fascinating to know that condensate clearance promotes plant disease tolerance (Tang *et al*., 2024). Herein, haematopoietic protein-1 (HEM1), containing a plant-specific condensation domain, binds to BI-1, further stabilizing the condensate and allowing ATG6 recruitment, ATG8 co-localization, and subsequent autophagic degradation. Thus, the removal of ER-associated HEM1 condensate formed upon pathogen stress (*Pseudomonas syringae* infection) is a plant defense strategy, as upon autophagy inhibition, plant lipid homeostasis is disturbed, leading to tissue damage (Tang *et al*., 2024). HEM1 and BI-1 condensates are a rich hub for lipid-metabolic enzymes, and it remains unknown if proteins, RNA, and lipids are the only biomolecular constituents of condensates. Future studies can resolve the composition and spatial/temporal distribution, as well as how possible modifications can influence condensate functions in plants.

This review focuses on recent research findings on biomolecular condensates associated with three organelles in the crosstalk between the plant endomembrane system and the autophagic pathway, namely the MVB, vacuole, and autophagosome. The autophagosome to MVB pathway mediates the fusion of autophagosomes with MVBs, forming an intermediate organelle, the ‘amphisome’ that fuses with the vacuole, delivering the cargo for degradation. In the MVB to vacuole pathway, the intraluminal vesicles (ILVs) containing membrane proteins and endocytosed materials enclosed within the MVBs are released into the vacuolar lumen upon MVB–vacuole fusion. Then, the autophagosome–vacuole pathway involves the bulk or selective engulfment of cytoplasmic cargoes within a growing phagophore, which transforms into a double-membraned autophagosome that fuses with the lytic vacuole for autophagic body delivery, degradation, and recycling. Here, we divided the autophagy pathway into early stage (autophagy initiation to phagophore maturation) and late stage (autophagosome formation and vacuolar fusion), highlighting their biomolecular condensate activities. In a holistic understanding of the association with these closely related and dynamically interacting organelles, we reveal outstanding questions in the field of plant cellular phase separation.

## Characterization methodologies and structural dynamics of plant biomolecular condensates

**Fig. 2.**
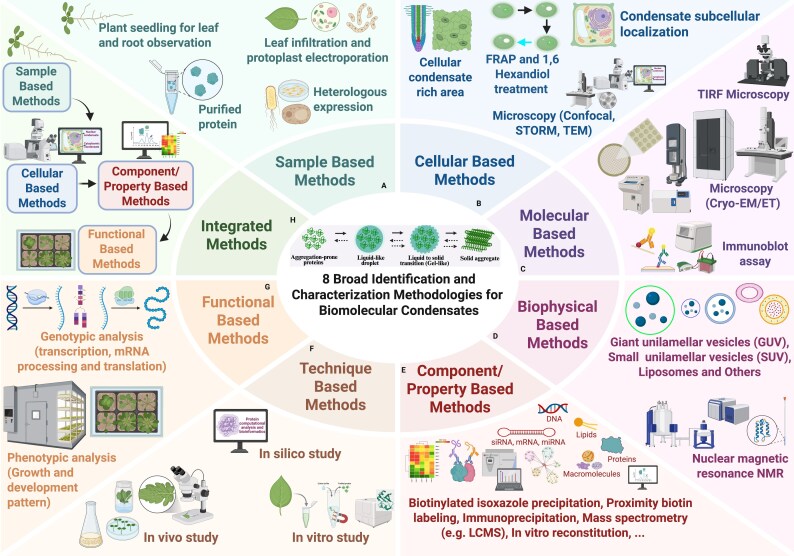
The eight broad identification and characterization methodologies for studying plant biomolecular condensates. (A) Sample-based methods involve the use of plant seedlings for leaf and root observation, leaf infiltration, and protoplast expression; heterologous expression and purified proteins reveal the biological system with an improved expression level for the target condensate-forming protein, providing an efficient system for fast testing of the full-length proteins and their domains for phase separation. (B) Cellular-based methods involve observing the plant condensate-rich region (shoot or root), FRAP analysis, 1,6-hexandiol treatment, condensate subcellular localization assay via confocal, super-resolution microscopy, and TEM imaging, measuring and detecting condensate formation (via disrupting LLPS), fluidity, viscosity, dynamics, subcellular localization, and structures. (C) Molecular-based methods using total internal reflection fluorescence microscopy (TIRFM), cryo-EM/ET, and immunoblot assay facilitate high-resolution single-molecule analysis and identification/quantification of target macromolecules and condensates. (D) Biophysical-based methods, involving the use of NMR, giant unilamellar vesicles (GUVs), small unilamellar vesicles (SUVs), liposomes, and other synthetically synthesized vesicles and membranes, provide information on the coordination, phase changes, and membrane–condensate interactions. (E) Component/property-based methods, involving biotinylated isoxazole precipitation, proximity biotin labelling, immunoprecipitation, LC-MS, and *in vitro* reconstitution, reveal the type and abundance of biomolecules present or interacting with the condensates. (F) Technique-based methods include the *in silico* study for a guided prediction and structural insights into the biomolecules, the *in vitro* study validating phase separation, and the *in vivo* studies confirming the physiological relevance of LLPS and the formed condensates. (G) Functional-based methods, involving phenotypic analysis (growth and developmental patterns) and genotypic analysis (transcription, mRNA processing, and translation), provide information about the relevance of the condensate presence or absence, its function, and possible future prospects that can be harnessed for a scale-up crop improvement. (H) Integrated methods reveal the importance of incorporating several methodologies to study plant biomolecular condensates. Created in BioRender. Mgbechidinma *et al*. (2025) https://BioRender.com/zr3a0nd.

Liquid-like condensates may undergo multiple transition processes during their conversion into more stable material states, such as the gel- and solid-like phases, marked by decreasing dynamics and reversibility (Fig. 1B). Recent advances in imaging, biochemistry, and genetic tools have enabled detailed identification and characterization of plant biomolecular condensate. We have further categorized these techniques into eight broad methods, namely sample-based methods, cellular-based methods, molecular-based methods, biophysical-based methods, technique-based methods, functional-based methods, component/property-based methods, and integrated methods (Fig. 2; Box 1). Owing to the fast germination rate and known genetic makeup of Arabidopsis and *Nicotiana* for easy experimental manipulation, these plant materials (seedlings or protoplasts) are widely used in the sample-based methods (Ouyang *et al.*, 2020; Kusumaatmaja *et al.*, 2021; Wang *et al.*, 2022; Dragwidge *et al.*, 2024; X. Li *et al*., 2024; Mosesso *et al.*, 2024; Tang *et al.*, 2024; Wang *et al*., 2024). Interestingly, in a recent protein phase transition study, the rice (*Oryza sativa*) natural variations of a *SIMILAR to RCD ONE* (*SRO*) gene, *OsSRO1c*, reveal an intrinsic LLPS ability, and confer cold tolerance at the seedling and booting stages (Hu *et al*., 2024).

Box 1. **Plant biomolecular condensate formation and its characterization/structural dynamics**

**Fig. 3.**
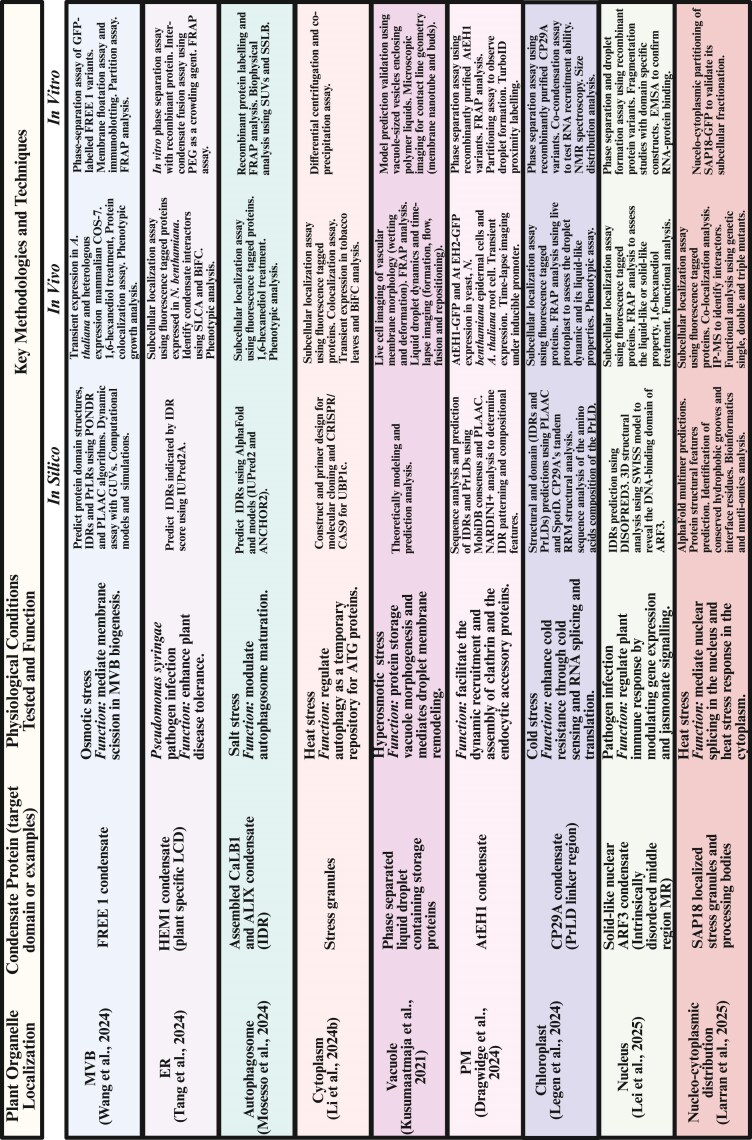
Summary of known condensate-forming proteins, functions, and key techniques in plants. The table highlights recent studies on plant organelle-localized condensates and the key technologies. ALIX, ALG2-interacting protein X; ARF3, AUXIN RESPONSE FACTOR 3; ATG, autophagy; BiFC, bimolecular fluorescence complementation assay; CaLB1, calcium-dependent lipid-binding protein 1; CP29A, chloroplast-localized RNA-binding protein 29A; ER, endoplasmic reticulum; FRAP, fluorescence recovery after photobleaching; FREE1, FYVE domain protein required for endosomal sorting 1; GUVs, giant unilamellar vesicles; HEM1, haematopoietic protein-1; IDR, intrinsically disordered region; LCD, low-complexity domain; MVB, multivesicular body; PEG, polyethylene glycol; PM, plasma membrane; PrLDs, prion-like domains; SAP18, Sin3-associated protein 18 kDa; SLCA, split-luciferase complementation assay; SSLB, solid-supported lipid bilayers; SUVs, small unilamellar vesicles; UBP1c, oligouridylate-binding protein 1c. Most of the studies also used confocal microscopy, TEM and immunogold labelling, electron tomography (ET), correlative light and electron microscopy (CLEM), stimulated emission depletion microscopy (STED), and conducted small-angle measurements. The condensate component and functional analysis were also analysed using immunoprecipitation–MS (IP-MS), ShinyGO for Gene Ontology analysis, split-luciferase complementation assay, co-localization assays, and inhibitor treatment assay. Created in BioRender. Mgbechidinma *et al*. (2025) https://BioRender.com/uf5r0hj.

- Owing to the non-reversibility and limited dynamics observed at the transition of aggregate-prone proteins (Fig. 1B), investigation into solid-like nuclear condensate could be harnessed for engineering gene expression and the nucleo-cytoplasmic translocation of proteins. A holistic condensate-based strategy controlled by a chemically inducible gene switch for transcription and translation can regulate the mammalian gene expression in a spatiotemporal manner (Wang *et al.*, 2025). This tool can be biotechnologically tethered for crop improvement.
- Major limitations in the methods illustrated in Fig. 2 include: (a) the transient and heterologous expression may lack relevant plant-specific components (biomolecules) and responses; (b) prolonged 1,6-hexanediol treatment can cause additional stress and induce granule formation, and the TEM samples need to be fixed, resulting in the loss of native structures and localization; (c) they require purified proteins, which are time-consuming and prone to contamination/degradation; (d) the biological samples need to be sufficient; (e) they are time-consuming and require the use of sophisticated equipment; (f) the use of bioinformatics tools requires skilled personnel; (g) they require control samples for validation; and (h) a combination of several limitations.
- Direct studies using Förster resonance energy transfer (FRET; a technique that measures how close two molecules are by observing the energy transfer between fluorescent labels) for plant biomolecular condensate studies are limited, but its principles suggest potential applications (Joshi *et al.*, 2024; Mgbechidinma *et al.*, 2025). In plants, biomolecular condensates are membrane-less structures where proteins, lipids, and RNA cluster via complex protein–protein and protein–RNA interactions. FRET can be engineered for condensate transition study to measure the proximity of labelled proteins within (detecting functional interactions), and monitor the dynamics of protein recruitment to or release from the condensates. Notably, the rapid formation and dissolution of plant biomolecular condensates and the sensitivity of FRET to environmental conditions (e.g. chlorophyll autofluorescence) are challenges to future studies.
- Biomolecular condensates and tethering serve as regulatory mechanisms for protein localization, with the nucleo-cytoplasmic distribution mediating signalling processes (Fig. 3). Besides the abiotic heat stress-induced nucleo-cytoplasmic partitioning reported for SAP18 (Larran *et al.*, 2025), another recent study demonstrated the stabilizing and accumulating role of ATG6 in regulating the distinct compartmentalization function of Nonexpressor of pathogenesis­related genes 1 (NPR1) in Arabidopsis (Zhang *et al.*, 2025), a well-characterized protein responsive to plant pathogen attacks and capable of forming condensate both in the nucleus and in the cytoplasm.

Cellular- and molecular-based methods emphasize the role of condensates in mediating endomembrane trafficking (Tang *et al.*, 2024; Wang *et al.*, 2024), alongside other pathways such as autophagy (Jiang *et al.*, 2024; Mosesso *et al.*, 2024; see Fig. 3). FRAP reveals condensate fluidity by measuring the fluorescence recovery rate, distinguishing liquid-like (rapid recovery) from solid-like (slow recovery) states (Fig. 1B). During cold acclimation in Arabidopsis, phase separation occurs in the presence of a PrLD in mNeonGreen-CP29A (chloroplast-localized RNA-binding protein CP29A), revealing a dynamic change within droplets upon FRAP analysis, with a fluorescence recovery half-life rate of 2.49±0.1 s for the full-length CP29A protein, 5.0±0.4 s for CP29A protein without the PrLD, and 2.5±0.1 s for PrLD alone (Legen *et al.*, 2024). The observed slow recovery rate in the absence of PrLD suggests a low protein mobility, which is indicative of a diminishing liquid-like property. Treatment with 1,6-hexanediol disrupts the hydrophobic interactions, as the condensate-localized VPS41 shows higher sensitivity compared with its state upon autophagy induction with menadione (oxidative stress treatment) in the presence of latrunculin B (LatB; an actin polymerization inhibitor) (Jiang *et al.*, 2024), emphasizing the function of 1,6-hexanediol in testing LLPS dependency. However, cautious interpretation of the results is required as prolonged 1,6-hexanediol treatment can induce artefactual aggregation. Super-resolution microscopy and TEM provide nanoscale resolution to reveal condensate morphology and subcellular localization. The protocol for the observation of membrane-less condensates in plant cells following high-pressure freezing-based EM coupled with immuno-gold labelling and correlative light electron microscopy (CLEM) techniques has been described (He and Li, 2024; Jiang *et al.*, 2024). Fluorescence microscopy using transient expression systems (e.g. protoplasts of Arabidopsis suspension cells or tobacco BY-2 cells) provides a rapid avenue for testing condensate-forming proteins, although the results may differ from endogenous studies due to overexpression artefacts. Stress granules, liquid-like condensates, and their transition phases in plants can also be observed using immunogold labelling and EM analysis to investigate autophagy induction, autophagosome–vacuole fusion, and liquid-phase ageing (Hutin *et al.*, 2023; Jiang *et al.*, 2024; X. Li *et al*., 2024).

Small-­angle X-ray scattering, X-ray diffraction, time-domain NMR (TD-NMR), and the use of giant or small unilamellar vesicles are known biophysical-based methods employed in condensate studies to mimic membrane curvature and membrane–condensate interactions (Hutin *et al.*, 2023; Legen *et al.*, 2024; Wang *et al.*, 2024). The component/property-based method is mainly reliant on multi-omics techniques to decipher the condensate macromolecules. Genetic approaches such as truncation, substitution, and mutation of IDRs and PrLDs through CRISPR, RNAi, and genetically encoded targeted protein degradation systems are vital in identifying sequences critical for condensation formation. The resultant outcomes are read through transcription, translation, and growth analysis, forming the basis of the functional-based methods. Interestingly, while a recent study showed that the removal of endogenous condensation-prone proteins (native TB1 protein and Early Flowering 3) improves crop performance (Luo *et al.*, 2025), several other studies demonstrated the positive regulation of condensate-forming proteins for plant growth, development, and stress adaptation (Wang *et al.*, 2022; Huang *et al.*, 2024; Legen *et al.*, 2024). Integrating coupled density gradient ultracentrifugation, quantitative MS, PICNIC (Proteins Involved in CoNdensates In Cells), as well as other predictive software is a high-throughput way in which to detect condensate-forming proteins (Hadarovich *et al.*, 2024; P. Li *et al*., 2024). While the *in vitro* methods (occurring in a controlled environment outside the living cell) and the *in vivo* methods (occurring within a living cell, tissue, or organism) are often incorporated in other methods, the technique-based methods also include the use of bioinformatic tools for predicting condensate-related proteins and their structures (Fig. 3). PrDOS (https://prdos.hgc.jp/cgi-bin/top.cgi), UniProt (https://www.uniprot.org/), Protein Data Bank (PBD https://www.rcsb.org/), PLAAC (http://plaac.wi.mit.edu/), PScore (https://github.com/haocai1992/PScore-online), IUPred2A (https://iupred2a.elte.hu/), and ESpritz (http://protein.bio.unipd.it/espritz/) predict the protein structures, the regions of multivalency, and the IDRs driving phase separation (Dragwidge *et al.*, 2024; Legen *et al.*, 2024; Mosesso *et al.*, 2024; Tang *et al.*, 2024; Wang *et al.*, 2024; Lei *et al.*, 2025). Condensate-forming proteins can be expressed and subsequently purified for *in vitro* reconstitution assay upon the addition of nucleic acids (RNA/DNA), salt at varying concentrations (as well as metal ions, such as manganese), ATP (energy source), and crowding reagents (Wang *et al.*, 2022; Poveda-Cuevas *et al.*, 2024). This allows for the investigation of the condensate substructure (shape and size), viscosity, fluidity, and spatiotemporal distribution via imaging approaches with FRAP and/or 1,6-hexanediol treatment, revealing the condensate kinetic state alongside the possibility of an integrated approach-based method.

The structural dynamics of plant biomolecular condensate start as dynamic, liquid-like structures, capable of fusion and component exchange, a process that can be assessed using differential interference contrast (DIC) imaging and FRAP. Upon changes in environmental cues or stress response, liquid-like structures transition to solid-like states (gelation), which can be analysed by small-angle X-ray scattering, X-ray diffraction, and particle tracking microrheology (Hutin *et al.*, 2023). Multivalency, driven by IDRs, facilitates phase separation, with viscosity and surface tension being commonly reported. These concepts mediate the selective sequestration or exclusion of condensate constituents, as observed between ATG proteins and stress granules (X. Li *et al*., 2024). Also, they induce membrane bending upon strong attractive forces (Wang *et al.*, 2024), and enable membrane molecular exchange, signal transduction, membrane tethering, and morphological regulation (Kusumaatmaja *et al.*, 2021; Subra *et al.*, 2023; Huang *et al.*, 2024; N. Kim *et al*., 2024; Poveda-Cuevas *et al.*, 2024; Wu *et al.*, 2024). Condensate morphology and intracondensate dynamics can be investigated via changing amino acid residues, as reported in the replacement of tyrosine with tryptophan (stronger) or phenylalanine (weaker) aromatic residues (Dorone *et al.*, 2021). Therefore, targeted amino acid mutation may regulate condensate formation in a dose-dependent manner, as revealed in the substitution of tyrosine residues for serine(s), leading to reduced LLPS (Dragwidge *et al*., 2024; Wang *et al*., 2024). Structural dynamics of biomolecular condensates are also influenced by molecular interactions, environmental cues, and conditions that control their formation, dissolution, transition, and ageing (Fig. 1A). Hybrid aspen ELF3 proteins form nuclear condensate and mediate low temperature-induced growth cessation through phase separation (Nair *et al.*, 2025), suggesting that temperature modulates condensate properties. LATE EMBRYO ABUNDANT (LEA) proteins, possessing an α-helical seed maturation protein (SMP) domain, mediate LLPS during seed desiccation (Ginsawaeng *et al.*, 2021). In addition, the nucleus-localized AUXIN RESPONSE FACTOR 19 (ARF19) transcription factor forms condensate in the cytoplasm where the C-terminal Phox and Bem1p (PB1) domain and the IDR-rich middle region (MR) drive protein multimerization and cytoplasmic condensate assembly, respectively (Górska *et al.*, 2023), implying that protein domains dictate phase behaviour. Conversely, the ARF19 condensate dissolves upon interacting with MULTIPLE C2 DOMAIN AND TRANSMEMBRANE REGION PROTEINS (MCTP3/4/6) at the lateral root, promoting nuclear localization of ARF (Xuan *et al.*, 2024). This underscores the role of the protein domain in balancing condensate stability and function in shaping plant root architecture. Notably, the structural transitions between liquid and gel states may reveal the adaptability property of condensates to cellular conditions. Challenges persist in linking condensate biophysics to its functions. Likewise, overexpression may alter condensate material properties, while *in vitro* assays may not recapitulate *in vivo* complexity. Future integration of live-cell imaging, cryo-EM/electron tomography/CLEM, and multi-omics techniques can refine our understanding of condensates and their regulation/functions in plant development and stress adaptation (Fig. 2).

## Crosstalk between the MVB, autophagosome, and vacuole

**Fig. 4.**
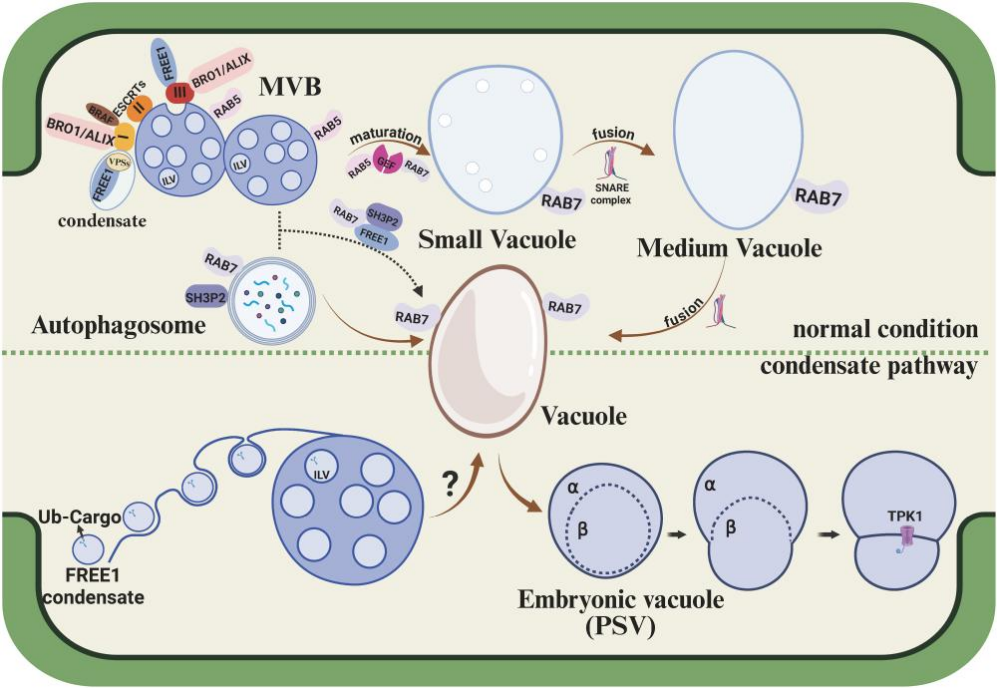
The endosomal–vacuolar trafficking through the ESCRT machinery and condensate-dependent pathway. In the typical MVB to vacuole and autophagosome to vacuole pathways, MVBs and autophagosomes deliver cargoes into the vacuole via direct membrane fusion, regulated by ESCRT machinery and Rab GTPases. As a plant-specific ESCRT component, FREE1 can form condensates with cargo, wetting and creating intraluminal vesicles (ILVs) within MVBs. Additionally, protein storage vacuoles (PSVs) can separate into two distinct phases, α and β, due to the presence of cargo and remodelling of new tonoplasts at the interface. ESCRT, endosomal sorting complex required for transport; FREE1, FYVE domain protein required for endosomal sorting 1; VPS, vacuolar protein sorting; GEFs, guanine nucleotide exchange factors; Rab, Ras-related protein; SH3P2, SH3 domain-containing protein2; and Ub, Ubiquitinated. Created in BioRender. Mgbechidinma *et al*. (2025) https://BioRender.com/xuyfhvu.

In plants, vacuoles are generally classified based on their functions into protein storage vacuoles (PSVs) and lytic vacuoles, which serve as primary sites for storage and metabolism, respectively. Lytic vacuoles contain hydrolytic enzymes that break down various biomolecules for recycling, making them the main catabolic compartments in plant cells. Before cargo is transported to the vacuole, it is typically sequestered into organelles such as autophagosomes or MVBs (Cui *et al*., 2018), indicating extensive dynamic interactions among these structures. The endosomal sorting complex required for transport (ESCRT) machinery, which includes ESCRT-0, ESCRT-I, ESCRT-II, and ESCRT-III, along with several accessory components, is evolutionarily conserved in these cellular processes (Fig. 4). Under normal conditions, soluble vacuolar cargo destined for degradation is recognized by vacuolar sorting receptor proteins and membrane proteins, with cargo internalized into the ILVs of MVBs by the ESCRT machinery. Rab GTPases, SNARE proteins, and other essential regulators facilitate the transition from MVBs to small vacuoles (SVs) and, subsequently, the fusion of SVs to form large and central vacuoles (Cui *et al*., 2019). Autophagosomes originate from the ER and deliver cargoes to vacuoles for degradation and recycling. Interestingly, the RAB7 GTPase and the plant-specific ESCRT component FREE1 have been observed on the membranes of both MVBs and autophagosomes (Gao *et al*., 2015). Besides its crucial role in MVB/vacuole biogenesis and cargo degradation, FREE1 is involved in autophagy through direct interaction with SH3P2, which binds to ATG8 and actively participates in autophagosome formation. In *free1* mutants, hybrid structures between autophagosomes and MVBs are also observed (Zhuang *et al*., 2015), suggesting a potential defect in the conversion process between these organelles.

## Condensate-mediated remodelling of the MVB and vacuole

In addition to the multiple functions of FREE1 ESCRT machinery in plants, recent research has revealed that the FREE1 protein contains IDRs and can effectively form condensates. FREE1 functions as a scaffold protein, interacting with ESCRT components such as VPS23 through its IDR and forming condensates to enhance the binding to membranes (Fig. 4). When the motif of FREE1 that interacts with VPS23 is removed, VPS23, along with its downstream components VPS27 and VPS38, is depleted from FREE1 condensates. Interestingly, variants of FREE1 that do not recruit VPS23 but retain phase separation ability can still rescue ESCRT defects, indicating that FREE1 plays a role in cargo sorting and degradation through a non-canonical ESCRT pathway (Wang *et al*., 2024). Through studies of condensate wetting morphologies and modelling, it was observed that mixing FREE1 condensates with giant unilamellar vesicles (GUVs) led to the formation of local membrane invaginations *in vitro*. These invaginations created vesicle-like structures filled with condensates and connected by membrane necks. Thus, the phase separation of FREE1, resulting in wetting interactions, facilitates the formation of ILV-like structures within MVBs (Wang *et al*., 2024) (Fig. 4). The ESCRT-mediated remodelling process for ILV formation and MVB biogenesis is largely conserved across yeast, animals, and plants. Most ESCRT components, such as VPS23 and VPS28, have highly homologous genes in yeast, humans, and plants (Weiner et al., 2025). However, FREE1 has uniquely evolved into an ESCRT protein specific to plants. In Arabidopsis, most ESCRT proteins contain IDRs, but *in vitro* experiments show that only FREE1 has the ability to form condensates (Wang *et al*., 2024). This raises the question of whether plants have evolved a distinct system for membrane curvature and scission. Interestingly, when FREE1 is ectopically expressed in mammalian cells, phase separation could also be observed, and both the number and size of ILV-like structures within MVBs significantly increase (Wang *et al*., 2024). This suggests that condensate-mediated MVB biogenesis also functions in non-plant systems. However, whether this mechanism is mediated by other key proteins in yeast and mammals or has been lost during evolution requires further investigation.

In plants, although FREE1 condensates can form freely diffusing vesicles without the aid of ESCRT machinery for membrane remodelling, it remains unclear whether a phase separation pathway exists during the fusion of MVBs into vacuoles. Moreover, phase separation and membrane wetting are crucial in the formation and morphogenesis of PSVs (Fig. 4). The cargoes within the PSVs facilitate the creation of micrometre-sized liquid droplets in the vacuolar lumen, which interact with the tonoplast to induce various membrane shapes, including buds and nanotubes. The interplay between membrane curvature and wettability determines the specific morphology, enabling the recruitment of new membrane proteins such as TPK1 between the α and β phases during budding, thus forming new tonoplast membranes (Kusumaatmaja *et al*., 2021). This highlights the role of intracellular wetting in organizing liquid phases and contributing to the droplet-mediated remodelling of vacuoles during seed development.

## Early plant autophagy stages and the roles of condensates

**Fig. 5.**
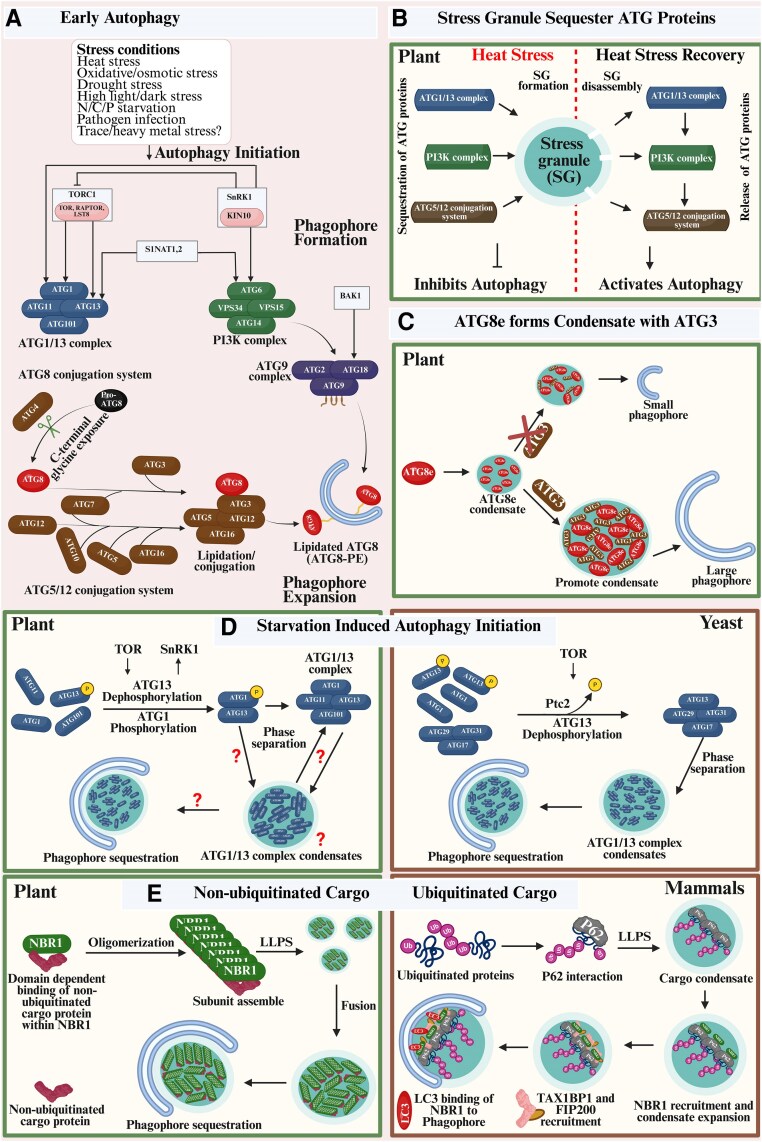
Biomolecular condensates as a platform for autophagy initiation and selective autophagy in plants. (A) The evolutionarily conserved autophagy pathway occurs at the basal level but can be induced under stress conditions. Schematic diagram of the early steps of plant autophagy. The ATG1/13 complex, ATG9 complex, and the PI3K complex initiate phagophore formation from the endoplasmic reticulum (ER). ATG9, together with ATG2 and ATG18, mediates phagophore expansion through lipid transfer and scramblases as the only transmembrane protein initially localized at the endosome or *trans*-Golgi network (TGN). Two ubiquitin-like conjugation systems [ATG7(E1) and ATG10(E2)] catalyse ATG12–ATG5 conjugation, while ATG7(E1), ATG3(E2), and ATG12–ATG5/ATG16 complex(E3) catalyse ATG8 lipidation. Lipidation in most ATG8 isoforms is preceded by the ATG4 cleavage that exposes the C-terminal glycine residue, enabling phosphatidylethanolamine (PE) covalent attachment and the subsequent incorporation of ATG8–PE into the autophagosome membrane. While TORC1, SnRK1, and SINAT are known regulators of autophagy, the plant hormone brassinosteroid (BR) signalling component ‘BAK1’ also plays an important role. (B) Upon heat stress recovery, the disassembly of stress granules and subsequent release of the ATG proteins activate autophagy. (C) ATG3 promotes autophagy by facilitating LLPS of ATG8e in Arabidopsis. (D) Condensate formation by IDR-rich complex ATG1/13 sets the stage for autophagy initiation upon nutrient starvation in yeast; however, whether phase separation occurs during ATG1/13 complex assembly remains to be explored in plants. (E) Ubiquitinated and non-ubiquitinated proteins are selectively degraded via condensate formation through the autophagy pathway in plants and mammals. ATGs, autophagy-related proteins; FIP200, focal adhesion kinase interacting protein of 200 kDa; LLPS, liquid–liquid phase separation; LC3, Microtubule-Associated Protein 1 Light Chain 3 (a functional homologue of plant ATG8); NBR1, Neighbor of BRCA1 gene 1; P, phosphorylation; Ptc2, PP2C phosphatases; SINAT, SEVEN IN ABSENTIA OF ARABIDOPSIS THALIANA; SnRK1, Sucrose-nonfermentation1-related protein kinase1; TAX1BP1, Tax1-binding protein 1; TOR, TARGET OF RAPAMYCIN; and TORC1, TOR complex 1. Created in BioRender. Mgbechidinma *et al*. (2025) https://BioRender.com/pg8wq57.

We termed the autophagy processes prior to the autophagosome maturation ‘early stages’ for a distinctive discussion of the involvement of LLPS and condensate formation (Fig. 5A). At the point of starvation-induced autophagy in yeast, biomolecular condensate is implicated during the cross-linking activity between the IDR-possessing ATG13 and the distinctly localized ATG17-binding and linking regions through LLPS to form a liquid-like PAS, aiding isolation membrane formation (Fig. 5D). Interestingly, the PAS is then tethered to the vacuolar membrane through interactions between ATG13 and the membrane protein Vac8, providing a platform for the recruitment of downstream autophagy factors and autophagosome formation (Fujioka *et al.*, 2020). While it is known that phosphorylation, as well as dephosphorylation, occurs at the ATG1/13 complex, with the ER being a well-characterized isolation membrane source during plant autophagy initiation, whether the bidirectional process [phosphoryl (PO_3_) group addition or removal] or the complex formation triggers biomolecular condensate formation remains unknown.

Biomolecular condensates formed by phase separation provide a reaction platform of wetting surfaces to sequester cargoes (Poveda-Cuevas *et al.*, 2024; Wang *et al.*, 2024). Bulk and selective autophagy are broadly studied in plants in response to biotic and abiotic stresses (Zhuang *et al*. 2024). Phase-separated condensates facilitate the interaction between cargoes and the autophagy machinery (Fujioka and Noda, 2021; Yan *et al.*, 2024; Licheva *et al.*, 2025). Proteins for recognizing and binding specific cargoes, namely selective autophagy receptors (SARs) as well as autophagy adaptors, assemble into phase-separated condensates to recruit specific targets (cargo) for selective autophagic degradation. Accordingly, the autophagy biogenesis factors phase-separate into initiation hubs at cargo surfaces in yeast and mammalian cells, creating multivalent, low-affinity interactions between the autophagy receptors and the cargo (Licheva *et al.*, 2025). SARs act like a molecular bridge that binds to the cargo while interacting with ATG8 in the autophagosome membrane via the ATG8-interacting motif (AIM) or the ubiquitin-interacting motif (UIM). An aromatic amino acid (tryptophan, tyrosine, or phenylalanine), followed by two random amino acids and an aliphatic amino acid (leucine, isoleucine, or valine), makes up the core AIM sequence (Rogov *et al.*, 2023; Licheva *et al.*, 2025). Moreover, the AIM aromatic residue interacts with the W pocket, while the aliphatic amino acid residue interacts with the L pocket. Both W and L are two distinct pockets of the AIM docking site in the ATG8 hydrophobic patch (Rogov *et al.*, 2023). Autophagy cargoes may include protein aggregates (aggrephagy), pathogens (xenophagy), or damaged organelles [lipid droplets (lipophagy), peroxisome (pexophagy), chloroplast (chlorophagy), ER (ER-phagy), and mitochondria (mitophagy)]. Examples of the ATG8-interacting SAR targeting protein substrates and complexes include (i) NBR1 for aggrephagy (X. Li *et al*., 2024 ; Yan *et al.*, 2024), (ii) dominant suppressor of KAR2 (DSK2) for BRI1-EMS suppressor 1 (BES1) in the brassinosteroid pathway (Han *et al.*, 2023), (iii) jasmonate-associated MYC2-like 1 (JAM1) in the jasmonate pathway (Zou *et al.*, 2023), (iv) regulator particle non-ATPase 10 (RPN10) for the 26S proteasome complex (Lin *et al.*, 2024), and (v) exocyst subunit EXO70B2 for the exocyst complex (De la Concepcion, 2023). This mechanistically differentiates selective autophagy from bulk autophagy, where randomly damaged cytosolic components are sequestered primarily upon nutrient starvation (Licheva *et al.*, 2025). Evidently, the Arabidopsis NBR1 (AtNBR1) also serves as a receptor for non-ubiquitinated cargo in biomolecular condensate-mediated autophagy (Fig. 5E) (Yan *et al.*, 2024). With more reports on an AtNBR1 homologue in mammals (P62 protein), known to mediate LLPS and condensate formation through the autophagy pathway (Turco *et al.*, 2021), researchers are yet to establish whether the sequestration of closely related organelles (by virtue of protein–protein or protein–lipid interactions evident in membrane contact sites) can be termed ‘selective autophagy’.

Stress granules, as plant cytoplasmic condensates, are also a possible source of AUTOPHAGY-related (ATG) proteins and facilitate ubiquitinated cargo sequestration upon heat stress recovery. RNA-binding protein 47B (RBP47B), poly(A)-binding proteins (PABs), oligouridylate binding protein 1 or uridylate (U)-rich RNA-binding protein 1 (UBP1; UBP1A, UBP1B, and UBP1C), and Tudor staphylococcal nucleases (TSNs) are known stress granule markers in plants (X. Li *et al*., 2024; Xie *et al*., 2024). During heat stress in Arabidopsis, ATG proteins accumulate and associate with stress granules in a manner that temporarily inhibits autophagy initiation (Fig. 5B). The release of the key autophagy components, including ATG1a/13a in the ATG1/13 complex, ATG6, VPS34 in the phosphoinositide 3-kinase (PI3K) complex, and ATG5 in the ATG8–phosphatidylethanolamine (PE) conjugation system during stress granule disassembly, enhances autophagy progression (X. Li *et al*., 2024). However, autophagy activation is delayed during the recovery phase in the *ubp1abc* mutant, which is deficient in assembly of stress granules, resulting in insufficient clearance of the heat stress-induced cargoes compared with the wild type (X. Li *et al*., 2024). Since stress granules are a temporary repository for key autophagy proteins during heat stress, the disruption in stress granule-mediated regulation of autophagy may lead to impaired plant stress recovery. The prion-like low complexity domain in RNA-binding proteins drives LLPS and facilitates stress granule assembly; further investigation into stress granule disassembly can reveal its reversible cytoplasmic nature. Aside from being reversible, stress granules are inducible and differ from P-bodies, which form constitutively (Xie *et al.*, 2024). Proteasomes are recruited during assembly and have been shown to control the disassembly of plant heat-induced stress granules, dictating tolerance upon stress recovery (Xie *et al.*, 2024). Autophagy-mediated clearance of heat stress-induced aggregates (ubiquitinated cargoes) occurs through sequestration by the AtNBR1 receptor (X. Li *et al*., 2024). As researchers delve into unveiling more selective autophagy-related receptors and adaptors, a recent report suggests that, beyond receptors and adaptors, starvation-induced autophagy can trigger the binding of non-SAR proteins (Pho81) to adaptors (ATG11) in an ATG13- and ATG17-independent manner to drive pexophagy in mammals (Gross *et al.*, 2024). Knowing that phosphorylation occurs upon stress induction and ubiquitination during cargo sequestration to the phagophore (Fig. 5A, D), future studies are needed to unveil the role of various plant protein PTMs in relation to LLPS, the valency, and strength of biomolecules interacting within the phase-separated condensates.

ATG8 and ATG3 have been shown to facilitate early phagophore maturation (Fig. 5C) through condensate-mediated progression during plant autophagy (Guan and Xue, 2022). Notwithstanding, knowledge about the compositional and functional plasticity of autophagy-related genes within each complex involved in autophagosome biogenesis and progression to the vacuole remains largely elusive in plant studies. Phase separation might provide a distinct mechanism to achieve metabolite microdomains in cells (Dumelie *et al.*, 2024). As such, future condensate profiling using omics techniques can be explored to understand the specificity and selectivity of cargoes during bulk/selective autophagy, as well as the fate of the autophagosome outer membrane after fusion.

## Biomolecular condensates upon autophagosome formation at the late autophagy stage

**Fig. 6.**
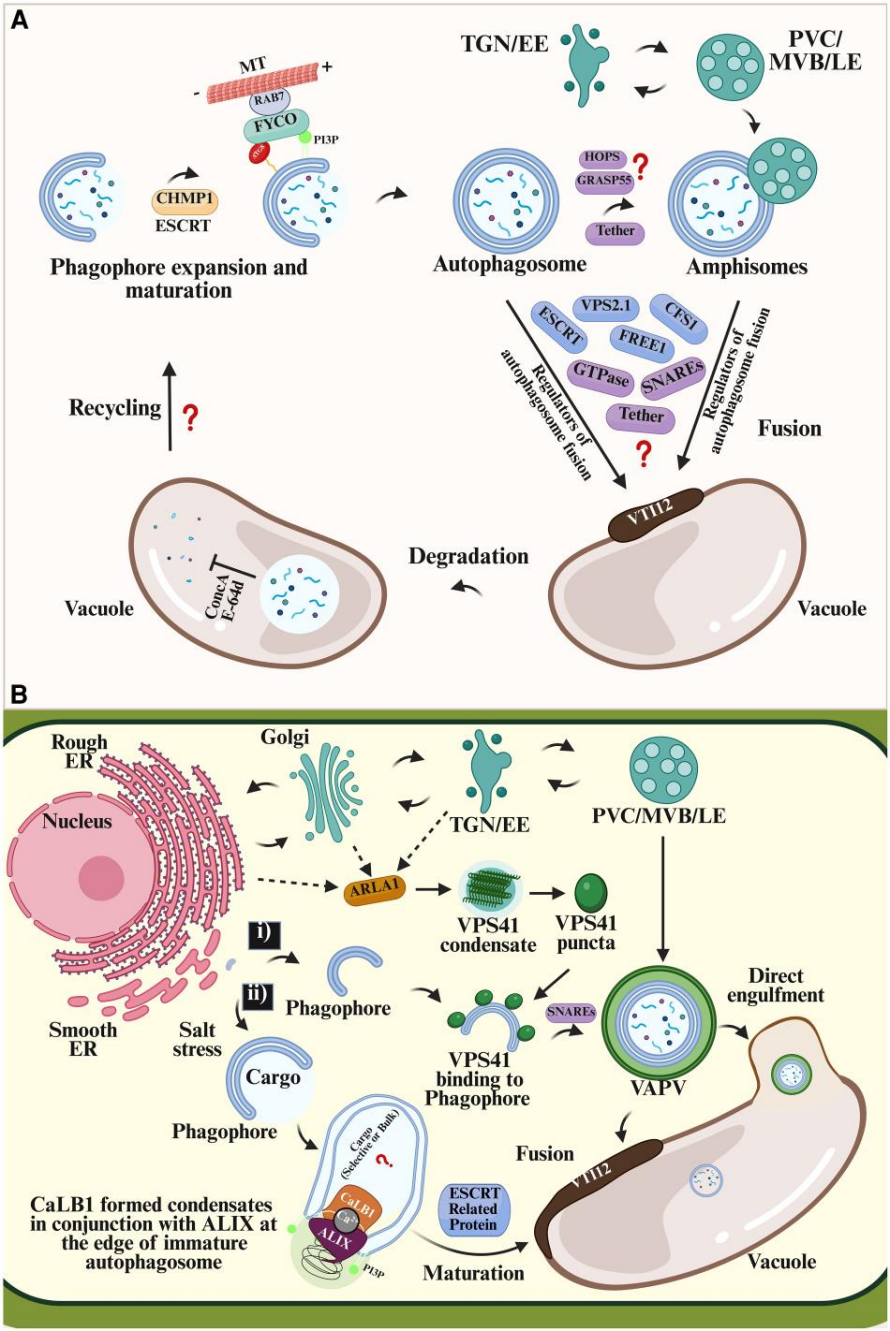
Autophagosome formation, trafficking, and vacuolar fusion require condensates. (A) Upon phagophore conjugation with lipidated ATG8 (ATG8–PE), an autophagosome as an enclosed double-membraned organelle is formed and transported to the vacuolar tonoplast for fusion via the microtubule (MT) cytoskeleton controlled by the ESCRT machinery and the v-SNARE-type mechanism. ESCRT mediates the phagophore closure to form an autophagosome. SNARE proteins, GTPase, and tethers mediate autophagosome–endosome (amphisome) and autophagosome–vacuole fusion. The released autophagic bodies are degraded within the vacuolar lumen, although the fate of the recycled components remains largely unknown. (Bi) ARLA1s regulate VPS41 LLPS-driven condensate formation under normal conditions and their dynamic conversion into VAPVs during autophagy. On losing liquidity, the condensate converts to puncta that decorate the expanding phagophore and, upon multivesicular body (MVB) fusion in a RAB7 GTPase independent manner, forms a VPS41-associated phagic vacuole (VAPVs), which completes the autophagy pathway through vacuolar degradation. (Bii) In a salt stress-specific condition, Arabidopsis Ca^2+^-dependent lipid-binding protein CaLB1 interacts with the ESCRT-associated protein ALIX at the autophagosome to undergo phase separation, facilitating the recruitment and positioning of ESCRT-III components for efficient closure and maturation of autophagosome. Created in BioRender. Mgbechidinma *et al*. (2025) https://BioRender.com/9ox3tn0.

Condensates, at and during autophagosome formation, raise questions on whether LLPS is the source of biomolecules required for the autophagosome closure, delivery, and fusion to the vacuole (Box 2). The Arabidopsis protein CaLB1, owing to its IDRs, drives the membrane curvature that provides large hydrodynamic radii to generate steric pressure, undergoes LLPS, and assembles phase-separated condensates of CaLB1 and ALIX *in vitro* and *in vivo* (Mosesso *et al.*, 2024). Upon salt stress-induced autophagy, CaLB1 and ALIX co-localize on the autophagosomes via ATG8 and PI(3)P, facilitating the recruitment and positioning of ESCRT-III components, which are crucial for the final membrane scission and closure of the autophagosome (Fig. 6Bii). This confirms the role of CaLB1–ALIX condensates in autophagosome maturation, as the disruption of CaLB1 function leads to an accumulation of unclosed autophagosomes, similar to the role of FREE1 which connects the ATG conjugation system and ESCRT-III complex in regulating autophagosome closure (Zeng *et al.*, 2023). However, whether the FREE1-mediated autophagosome closure requires condensate formation has yet to be established.

Box 2. **Early and late plant autophagy stages and the roles of condensates**

- Recent studies revealed the close relationship of plant autophagy with its associating condensates, which are formed through LLPS of biomolecules such as proteins, nucleic acids, and lipids, that are assembled into liquid-like droplets at the basal level due to the presence of prone proteins or upon response to environmental changes such as salt and heat stresses.
- Figure 5A: changes from favourable environmental conditions and nutrient deprivations (stresses) have been reported to trigger autophagy and biomolecular condensate formation. Signalling molecules, such as kinases and phosphatases, can concentrate within phase-separated condensates to modulate the activity and localization of ATG proteins. These regulatory condensates integrate various stresses and nutrient signals to coordinate the early stages of the autophagy process, supporting the fact that biomolecular condensates are a principal regulator of autophagy.
- Figure 5B: stress granules transiently sequester ATG proteins during heat stress in Arabidopsis, which delays autophagy activation until the stress granule disassembles post-stress upon recovery, thus enabling rapid degradation of the ubiquitinated protein aggregates (X. Li *et al*., 2024). This dynamic regulatory hub leverages LLPS to spatiotemporally coordinate autophagy activation, thereby balancing stress survival and proteotoxic damage clearance in plants.
- Figure 5C: the autophagy marker ATG8, especially its isoform ATG8e in plants, can be phase separated using its N-terminal IDR, and the condensate is promoted by ATG3 *in vitro* and *in vivo* (Guan and Xue, 2022). The atg3ΔIDR mutant had fewer liquid condensates than the wild type, indicating that ATG3 affects ATG8e phase separation and thus regulates autophagy.
- Figure 5D: starvation is known to induce autophagy in plants; however, whether phase separation occurs during the assembly of the ATG1/13 complex remains unknown. The autophagy-initiating ATG1/13 complex contains several IDRs in plants and yeasts. The ATG1/13 complex in yeast undergoes LLPS to form dynamic liquid droplets *in vitro* through specific multivalent interactions between the ATG13 and ATG17 subunits of the complex (Fujioka *et al*., 2020).
- Figure 5E: cargo sequestration is a known phenomenon in the autophagy process. Yan *et al*. (2024) showed that non-ubiquitinated cargo in plants can be sequestered to the phagophore upon condensate formation. In mammals, the roles of p62, NBR1, and TAX1BP1 in ubiquitin condensate formation and autophagy initiation have been well documented following a reconstitution assay (Turco *et al*., 2021).
- Figure 6B (i): VPS41 condensate to VAPV formation through contact with a developing phagophore that enhances autophagosome delivery to the vacuole occurs in a RAB7 GTPase-independent manner (Jiang *et al*., 2024).
- Figure 6B (ii): salt-induced condensates, formed by calcium-dependent lipid-binding protein 1 (CaLB1) and ALG2-interacting protein X (ALIX) on immature autophagosome, facilitate closure and maturation through subsequent recruitment of the ESCRT machinery (Mosesso *et al*., 2024).

To date, little is known about the components that facilitate autophagosome delivery. Although not experimentally proven in plants, phase-separated condensates may help regulate the localization and interactions of the cytoskeletal components involved in the trafficking and fusion of autophagosomes with the vacuoles for cargo degradation and recycling (Fig. 6A). Recently, Jiang *et al*. (2024) demonstrated that the conversion of VPS41 condensates into puncta and then into ring-like VAPVs (VPS41-associated phagic vacuoles) is essential for the proper trafficking and transport of autophagosomes to the vacuole for degradation. Disruption of this condensates-to-VAPVs conversion process (by mutating either the upstream regulators ARLA1s or the key autophagy genes) impairs the vacuolar transport of autophagosomes, leading to their accumulation outside the vacuole. The ring-like acidic structure VAPVs formed from the VPS41 condensates appear to act as specialized carriers or transport intermediates that escort autophagosomes to the vacuole, like the autophagy marker ATG8 localized within the structure (Fig. 6Bi), suggesting its role in coordinating the autophagy membrane dynamics and trafficking. VPS41, a subunit of the conserved HOPS complex, is localized not only to the tonoplast (vacuolar membrane) but also to distinct punctate condensates in plant root cells. Its formation does not rely on AP-3 or Rab7 pathways, known as upstream vacuolar transport regulators, as condensates persist in the absence of the HOPS complex (Jiang *et al.*, 2022). The liquid-like properties of the condensates facilitate fusion and fission. Therefore, VPS41 condensates may coordinate activities between the ER and vacuoles, as its closely associated organelles, indicating a plant-specific function for VPS41, independent of its role in the HOPS complex for homotypic vacuole fusion. While the low pH level of the VAPVs is indicative of their priming function at the late stages of autophagy, it is not known if the degradative process starts within the VAPVs upon MVB fusion or after vacuolar fusion/engulfment. It will be vital to know if this specialized condensates-to-VAPVs conversion pathway is a plant-specific regulatory autophagy process, highlighting the importance of biomolecular phase separation in the dynamic organization and progression of plant quality control. Hence, the VPS41 condensates-to-VAPVs may be a pathway to uncover the mystery in the delivery of cargoes, especially larger organelles, through the autophagosome to the vacuolar lumen for degradation and recycling.

## Conclusions and outstanding questions

Although phase separation studies in the context of plant physiology, and molecular and cellular biology will continue to grow, studies in yeast and mammals are more advanced. As we have yet to understand fully the intricacy of condensates, recent studies have shown that LLPS regulates various important cellular processes in plants. These include transcription, miRNA processing, phytohormone sensing, adaptation to higher temperatures, cargo sorting, pathogenic responses, circadian rhythms, meiotic transition timing, and seed germination. Albeit this review focuses on MVBs, autophagosomes, and vacuoles, forming biomolecular condensates through LLPS provides a mechanism for spatially organizing and concentrating specific components within the plant cell to facilitate these diverse physiological functions. The dynamic and liquid-like nature of these phase-separated compartments allows for rapid responses to changing environmental and developmental cues in plants. Understanding how cells organize and compartmentalize their interiors is key to elucidating how they function and respond to changes. Possible classification criteria of plant condensates could include their localization, protein mobility, size, enzyme activity, and specific protein or RNA recruitment alongside other heterogeneous properties. While harnessing these potentials could enhance the transformative output of research findings to fieldwork, there are still many open questions despite the significant progress in studying plant endomembrane organelles and biomolecular condensates. Therefore, delving into the compositional and structural complexity of various types of condensates would provide a better clue to the origin of their heterogeneity. Future collaborative research efforts are required to investigate how these condensates influence membrane shape, interact with different organelles, and control the movement of molecules within the cell. Considering the dynamic interaction among different stress-related condensates, such as the stress granules and P-bodies, one can only wonder what constituent makes a particular condensate large and the other small at a specific time. For example, do condensates undergo fusion and/or fission events, or are amphipathic proteins known for size regulation in lipid bodies also integral condensate constituents? A deeper understanding of plant biomolecular condensates can offer new insights into plant cell and molecular biology, and may lead to new agricultural strategies for crop improvement and resilience through condensate engineering.

Outstanding questions and future experiments in the growing field of plant biomolecular condensate formation include the following.

- Given the correlation in adhesion, membrane elasticity, and interfacial tension between condensates and membranes, does LLPS at different endomembrane organelles differ? Can the formed condensates be manipulated by introducing the key biomolecules to induce or removing them to abolish condensate formation? What are the origin, structure, and evolutionary mechanisms of membrane-less organelles in plant cells?
- Condensates are found in close proximity to several protein complexes and organelles (e.g. ER, phagophore, autophagosome, endosome, and vacuole) at different stages of the autophagy pathway. It is important to know if these organelles undergo phase separation independently of the ATG proteins or if it is an intrinsic part of the autophagy pathway. Is the formation and disassembly of biomolecular condensates as highly regulated as the canonical autophagy pathway, or are the condensates transiently involved in the autophagy process? Suppose condensate is closely related to the MVB, autophagosome, and vacuole, mostly involved in plant quality control, does this imply that phase-separated proteins can determine the timing of survival as a redundant mechanism?
- In a controlled biophysical reconstitution experiment, LLPS interactions can be studied at near-physiological conditions using a supported lipid bilayer (SLB) and other tools such as GUVs, giant plasma membrane vesicles (GPMVs), or a double supported lipid bilayer (DSLB). These methods have restricted geometry; therefore, biochemical reconstitution experiments may not accurately reflect cell condensate behaviour, suggesting a need for improvement or modification of techniques similar to live cells.
- How are findings from *in vivo* and *in vitro* assays (e.g. condensate reconstitution) compared? Despite the fact that condensate structures can be resolved by TEM (Jiang *et al.*, 2024), super-resolution live-cell imaging techniques are required in investigating the dynamic assembly of condensates in living cells, allowing for more dynamic studies and examination of their viscoelastic properties.
- Liquidity is a critical determinant for selective autophagy of protein condensates. Does the organelle source of the isolation membrane provide any hint for the possibility of condensate formation or the sequestration of bulk or selective cytoplasmic constituents?
- With the enrichment of condensates with phospholipids (Dumelie *et al.*, 2024), does LLPS drive the formation of phospholipids, their precursors, and interaction with ATG8 within the autophagy pathway. If this is the case, can the presence or need for phospholipid be a marker for possible condensate formation during autophagy? As membrane-less organelles, condensates can be easily envisaged as not forming lipid bilayers; however, what is the specific conformation and spatial orientation of lipids within the condensates?
- Similar to yeast, where the cytoplasm-to-vacuole targeting pathway is established, does ubiquitin-independent autophagic targeting of semi-liquid/gel-like biomolecular condensates to the vacuole occur in plants?
- Green fluorescent protein (GFP)-tagged proteins expressed in plant cells under the constitutive promoters (35S or UBQ) often form aggregates as detected by confocal imaging. They are often considered as ‘overexpression artefacts’. With more evidence for the detection and function of LLPS in plants, we should be more careful to distinguish the ‘artefacts’ and the physiologically functional LLPS in future plant biology research.
